# Iron Deprivation Affects Drug Susceptibilities of Mycobacteria Targeting Membrane Integrity

**DOI:** 10.1155/2015/938523

**Published:** 2015-12-08

**Authors:** Rahul Pal, Saif Hameed, Zeeshan Fatima

**Affiliations:** Amity Institute of Biotechnology, Amity University Haryana, Gurgaon, Manesar 122413, India

## Abstract

Multidrug resistance (MDR) acquired by* Mycobacterium tuberculosis* (MTB) through continuous deployment of antitubercular drugs warrants immediate search for novel targets and mechanisms. The ability of MTB to sense and become accustomed to changes in the host is essential for survival and confers the basis of infection. A crucial condition that MTB must surmount is iron limitation, during the establishment of infection, since iron is required by both bacteria and humans. This study focuses on how iron deprivation affects drug susceptibilities of known anti-TB drugs in* Mycobacterium smegmatis*, a “surrogate of MTB.” We showed that iron deprivation leads to enhanced potency of most commonly used first line anti-TB drugs that could be reverted upon iron supplementation. We explored that membrane homeostasis is disrupted upon iron deprivation as revealed by enhanced membrane permeability and hypersensitivity to membrane perturbing agent leading to increased passive diffusion of drug and TEM images showing detectable differences in cell envelope thickness. Furthermore, iron seems to be indispensable to sustain genotoxic stress suggesting its possible role in DNA repair machinery. Taken together, we for the first time established a link between cellular iron and drug susceptibility of mycobacteria suggesting iron as novel determinant to combat MDR.

## 1. Introduction

Tuberculosis (TB) caused by* Mycobacterium tuberculosis* (MTB) continues to pose significant global health challenges that require immediate treatment regimens directed at new targets. TB is remediable; however, due to its long course of medication or mismanagement in drug regimen, it has led to the emergence of multidrug resistance tuberculosis (MDR-TB) against various frontline anti-TB drugs [1–3]. Despite reasonable documentation of major factors which contribute to MDR mechanisms, it appears unavoidable to dissect novel mechanisms combating MDR [4]. Iron deprivation represents one of the crucial environmental stress conditions that MTB encounters during infection process due to nonavailability of free iron in human host [5, 6]. Availability of iron in host cells is therefore tightly regulated making it less available to both the host and the invading pathogen like MTB. Thus, targeting the iron homeostasis could be one of the strategies that could be efficiently adopted to impede the fast growing resistance.

The role of iron in drug susceptibility has already been established in other major human pathogens, namely,* Candida albicans*,* Leishmania donovani*,* Staphylococcus aureus*, and* Streptococcus epidermidis* [7–11]. It has been showed that iron depletion in* Candida albicans* with bathophenanthroline disulfonic acid (BPS) and ferrozine as chelators increased its sensitivity to many common antifungal drugs, including fluconazole (FLC). Many different species of* Candida* also showed an increase in the drug susceptibility under iron limitation. The effect of iron chelation on the growth of* Leishmania (Viannia) braziliensis*, expression of proteins, and ultrastructure of this parasite has also been studied. Similar study on Gram-positive bacteria* Staphylococcus* was done in the presence of iron chelator diamine diorthohydroxyphenyl acetic acid.

In mycobacteria, a link between phospholipid homeostasis, virulence, and iron acquisition has been recently explored by lipidomic approach [12]. The antimycobacterial activities of pyrazolopyrimidinone and ATP have also been attributed to their iron chelating abilities [13, 14]. Even the various iron acquisition strategies have been reviewed to understand the potential of few iron dependent candidates and protein which may influence the elimination of mycobacteria from the host [5, 15, 16]. Thus, the significance of iron in mycobacteria is emerging and also well established as apparent from a wide range of recent studies. However, no such direct study depicting the link between iron and drug susceptibility of mycobacteria has yet been experimentally demonstrated.

The objective of the present study was to find out a correlation between iron availability and drug susceptibility of mycobacteria to known anti-TB drugs. In this study, for the first time, the role of iron in governing the drug susceptibility of known anti-TB drug is explored. We showed that iron deprivation leads to enhanced potency of first line anti-TB drugs (ethambutol, isoniazid, and rifampicin) that could be reverted upon iron supplementation. We explored that iron deprivation leads to disrupted membrane homeostasis which was confirmed by enhanced membrane permeability and hypersensitivity to membrane perturbing agent and TEM images. We also showed that iron deprivation leads to enhanced genotoxicity in the presence of ethidium bromide suggesting its possible role in DNA repair mechanisms. Together, this study revealed an intricate relationship between cellular iron and drug susceptibility of mycobacteria.

## 2. Materials and Methods

### 2.1. Materials

All Media chemicals Middlebrook 7H9 broth, Middlebrook 7H10 agar, albumin/dextrose/catalase (ADC), and oleic acid/albumin/dextrose/catalase (OADC) supplements were purchased from BD Biosciences (USA). Deferoxamine mesylate salt powder (DFO), bathophenanthroline disulfonic acid disodium salt (BPS), Tween-80, nitrocefin, ethambutol (EMB), and isoniazid (INH) were purchased from Sigma-Aldrich (St. Louis, MO, USA). 2,2, Bipyridyl (2,2, BP), ethidium bromide (EtBr), dinitrophenol (2,4, DNP), and rifampicin (RIF) were purchased from Himedia (Mumbai, India). Dimethyl sulfoxide (DMSO), potassium chloride (KCl), sodium chloride (NaCl), disodium hydrogen orthophosphate (Na_2_HPO_4_), potassium dihydrogen orthophosphate (KH_2_PO_4_), sodium dodecyl sulphate (SDS), glycerol, and D-glucose were obtained from Fischer Scientific; methanol was purchased from Merck.

*Bacterial Strains and Culture Conditions. M. smegmatis* mc^2^155 was grown in Middlebrook 7H9 (BD Biosciences) broth supplemented with 0.05% Tween-80 (Sigma), 10% albumin/dextrose/catalase (ADC; BD Difco), and 0.2% glycerol (Fischer Scientific) in 100 mL flasks (Schott Duran) and the culture was incubated at 37°C and on Middlebrook 7H10 (BD Biosciences) agar media supplemented with 10% (v/v) oleic acid/albumin/dextrose/catalase (OADC; BD Difco) for solid agar allowing growth for 48 h at 37°C. Stock cultures of log-phase cells were maintained in 30% glycerol and stored at −80°C.

### 2.2. Drug Susceptibility Testing

#### 2.2.1. Minimum Inhibitory Concentration (MIC)

MIC was determined by broth dilution method described elsewhere [17] according to the guidelines of CLSI [18]. Briefly, 100 *μ*L of Middlebrook 7H9 broth was placed at each well of the 96-well plate following with the addition of the drug with the remaining media and then subsequently it was serially diluted 1 : 2. 100 *μ*L of cell suspension (in normal saline to an O.D_600_ 0.1) was added to each well of the plate. Plates were incubated at 37°C for 48 hours. The MIC values were evaluated by observing the O.D_600_ in a microplate reader (Lisa Reader). The MIC_80_ was defined as the concentration at which 80% of the growth was inhibited compared with the controls.

#### 2.2.2. Spot Assay

Spot assays for the strains were determined using a method as described elsewhere [19, 20]. Briefly, for the spot assay, 5 *μ*L of fivefold serial dilutions of each* M. smegmatis* culture (each with cells suspended in normal saline to an O.D_600_ nm of 0.1) was spotted onto Middlebrook 7H10 agar plates in the absence (control) and the presence of the drugs. Growth difference was measured after incubation at 37°C for 48 hours.

#### 2.2.3. Membrane Permeability Assay

The *β*-lactamase activity for the permeabilization of* M. smegmatis* was determined by measuring the hydrolysis of nitrocefin by whole cells as described elsewhere [21, 22]. Briefly, cells were grown overnight at 37°C in the absence (control) and the presence of 2,2, BP at its subinhibitory concentration with continuous shaking. Cells were then equalized with cold 1X phosphate-buffered saline (PBS) buffer (pH 7.4). Nitrocefin was added at a final concentration of 0.25 mg/mL to the aliquot of cells (2 mL) in 1X PBS (pH 7.4), and hydrolysis was monitored as a change in absorbance at 486 nm till 60 min using a double beam spectrophotometer (VSI-501).

#### 2.2.4. Transmission Electron Microscopy (TEM)

Treated and untreated cells of* M. smegmatis* cells were observed using TEM (JEOL JEM-1011). The cells of 0.1 O.D_600_ were seeded to the media with and without drugs and were incubated for 24 h at 37°C. Sample preparation and analysis were done by using the method as described elsewhere [23]. Briefly, cells were harvested in phosphate-buffered saline (PBS) fixed with 2.5% glutaraldehyde in 0.1% phosphate buffer for 1 h at room temperature (20°C), washed with 0.1 M phosphate buffer (pH 7.2), and postfixed with 1% OsO_4_ in 0.1 M phosphate buffer for 1 h. Cells were then dehydrated through ethanol, dried and coated with gold, and observed at magnification of 15000X.

#### 2.2.5. Passive Diffusion of Drug

The diffusion of EtBr was determined by using protocol described elsewhere with modification [17, 24]. Briefly, cells were grown till exponential phase in the absence (control) and in the presence of iron deprived condition (2,2, BP). Cells were pelleted, washed twice with phosphate-buffered saline (PBS), and resuspended as a 2% cell suspension. The cells were then deenergized with an efflux pump inhibitor 2,4, DNP (20 *μ*g/mL) in PBS to block the functionality of efflux pumps. The deenergized cells were pelleted, washed, and again resuspended as a 2% cell suspension (w/v) in PBS, to which EtBr was added at a final concentration of 4 *μ*g/mL and incubated for 45 min at 25°C. The equilibrated cells with EtBr were then washed and resuspended as a 2% cell suspension (w/v) in PBS. Samples with a volume of 2 mL were withdrawn at the indicated time points and centrifuged at 10,000 rpm for 1 min. The supernatant was collected and absorption was measured at 285 nm.

## 3. Result and Discussion

### 3.1. Assessment of Mycobacterial Growth in Response to Iron Deprivation

**Figure 1:**
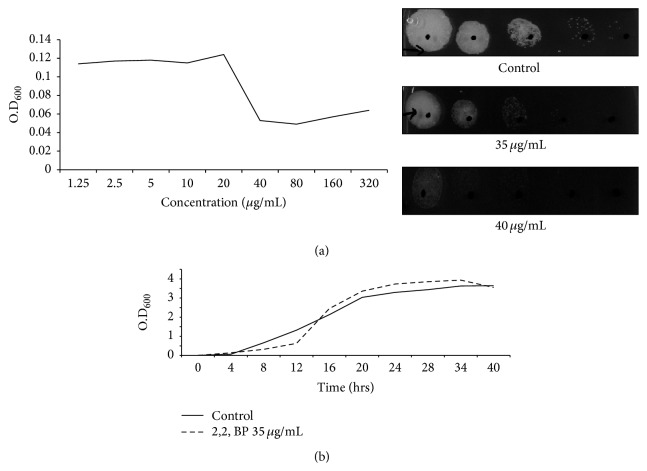
Assessment of mycobacterial growth in response to iron deprivation. (a) Drug susceptibility assays against* M. smegmatis* in the presence of 2,2, BP. Left panel shows broth microdilution assay to determine the MIC_80_ of* M. smegmatis* in the presence of 2,2, BP. The minimum drug concentration that inhibits growth by 80% relative to the drug-free growth control is indicated as MIC_80_. Right panel shows spot assay of* M. smegmatis* in the absence (control) and the presence of subinhibitory concentration of 2,2, BP (35 *μ*g/mL). (b) Growth curve of* M. smegmatis* in the presence of 35 *μ*g/mL 2,2, BP.

It is known that iron deprivation affects the growth of several microorganisms including mycobacteria [5]. Therefore, before proceeding with any experiment, we needed to rule out such concerns by assessing the growth of* M. smegmatis* cells and demonstrated that while 2,2, BP was sufficient to chelate iron at the concentration used in this study, it did not affect the growth of the cells. Thus, the growth of* M. smegmatis* cells was evaluated in the presence of 2,2, BP, a well-known iron chelator, at a concentration subsequently used in the study. To find out whether iron depletion leads to any growth defect was achieved by two different methods, namely, broth microdilution and spot assays.  Figure 1(a) illustrates that the growth of* M. smegmatis* was completely inhibited at 40 *μ*g/mL 2,2, BP; however, growth was not affected appreciably when cells were grown at 35 *μ*g/mL 2,2, BP. This subinhibitory concentration was also confirmed by growth curve performed in the absence and the presence of 35 *μ*g/mL 2,2, BP (Figure 1(b)). These results ensure that the concentration of 2,2, BP higher than 35 *μ*g/mL caused growth inhibition and hence cannot be used for further experiments.

### 3.2. Iron Depletion Makes* M. smegmatis* More Susceptible to First Line Anti-TB Drugs

**Figure 2:**
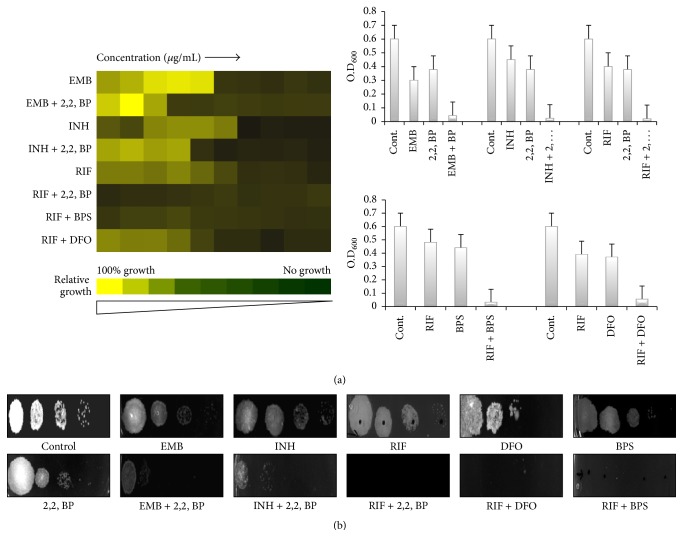
Drug susceptibility assays against* M. smegmatis* in response to iron deprivation. (a) Broth microdilution to determine the MIC_80_ of* M. smegmatis* in the presence of EMB, INH, and RIF (62.5 ng/mL, 1 *μ*g/mL, and 62.5 ng/mL) alone and in the presence of 2,2, BP, DFO, and BPS (35 *μ*g/mL, 656 *μ*g/mL, and 386 *μ*g/mL), respectively, to determine the effect of iron deprivation on susceptibilities of EMB, INH, and RIF. Data was quantitatively displayed with color (see color bar in left panel), where each shade of color represents relative optical densities of the cell and as bar graphs (see right panel). The minimum drug concentration that inhibits growth by 80% relative to the drug-free growth control is indicated as MIC_80_ for each drug. (b) Spot assays of* M. smegmatis* in the presence of EMB, INH, and RIF (62.5 ng/mL, 1 *μ*g/mL, and 62.5 ng/mL) alone and in the presence of 2,2, BP, DFO, and BPS (35 *μ*g/mL, 656 *μ*g/mL, and 386 *μ*g/mL) to confirm the effect of iron deprivation on susceptibilities of EMB, INH, and RIF.

Broth microdilution and spot assays were used to find out whether iron depletion leads to any changes in drug susceptibilities of* M. smegmatis*. Firstly, we studied the drug susceptibility of three different classes of known anti-TB drugs (EMB, INH, and RIF) without any iron deprivation. We found that MIC_80_ for above drugs alone were observed at 0.25 *μ*g/mL, 4 *μ*g/mL, and 2 *μ*g/mL, respectively (Figure 2(a)). Interestingly, when the cells were deprived of iron due to the presence of 2,2, BP, the sensitivity for all of the anti-TB drugs (EMB, INH, and RIF) tested was further enhanced to 62.5 ng/mL, 1 *μ*g/mL, and 62.5 ng/mL, respectively. Spot assays also revealed that cells in iron deprived condition (2,2, BP) were distinctly more susceptible to EMB, INH, and RIF compared to those growing under iron sufficient conditions (Figure 2(b)). Growth was not affected by the presence of respective solvents of drugs used in the examination (data not shown).

To confirm whether the observed enhanced drug susceptibility of* M. smegmatis* cells is not chelator specific property and is due to iron limitation only, we perform similar susceptibility assays in the presence of other well-known iron chelators DFO (656 *μ*g/mL) and BPS (368 *μ*g/mL) at their subinhibitory concentrations. MIC_80_ results showed that cells grown in the presence of either DFO or BPS showed increased sensitivity as compared to that of those grown under iron sufficient conditions (Figure 2(a)). MIC_80_ results were also confirmed by spot assays (Figure 2(b)), which clearly depicts enhanced susceptibilities under iron deprivation. Thus, we established that deprivation of iron resulted in enhanced sensitivity of* M. smegmatis* cells to most of the commonly used known first line anti-TB drugs.

### 3.3. Iron Supplementation Reverses the Enhanced Susceptibilities of* M. smegmatis* to Anti-TB Drugs

**Figure 3:**
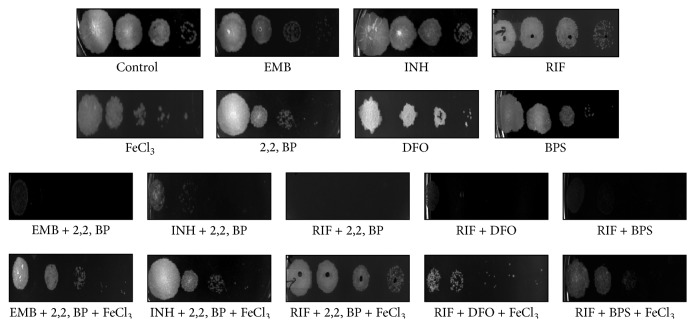
Effect of iron supplementation on reversion of drug susceptibilities. Spot assays of iron deprived cells (2,2, BP, DFO, and BPS) at concentrations of 35 *μ*g/mL, 656 *μ*g/mL, and 386 *μ*g/mL showing hypersensitivity with EMB, INH, and RIF and upon iron supplementation (FeCl_3_) of 0.2 mM for 2,2, BP, 0.3 mM for BPS, and 0.4 mM for DFO showing rescuing of the drug susceptibilities of EMB, INH, and RIF (62.5 ng/mL, 1 *μ*g/mL, and 62.5 ng/mL).

To further confirm whether the enhanced drug susceptibilities of* M. smegmatis* cells observed are due to iron limitation only, we perform spot assays by supplementation of iron back to the media. Remarkably, when these cells were grown in the presence of FeCl_3_, the enhanced susceptibility of all the three drugs (EMB, INH, and RIF) tested in iron deprivation could be rescued (Figure 3). Of note, since different iron chelators have their own specific iron binding abilities, different concentrations of FeCl_3_ were used to rescue the growth. Thus, a direct link between iron levels and drug susceptibility was further established when the drug-sensitive phenotype was found to be reversed upon supplementation of the growth media with iron. Our results reinforced the fact that iron does play a crucial role in enhancing the drug susceptibilities in* M. smegmatis* cells and its mechanism of action needs to be worked out.

### 3.4. Iron Deprivation Affects Membrane Homeostasis of* M. smegmatis*

**Figure 4:**
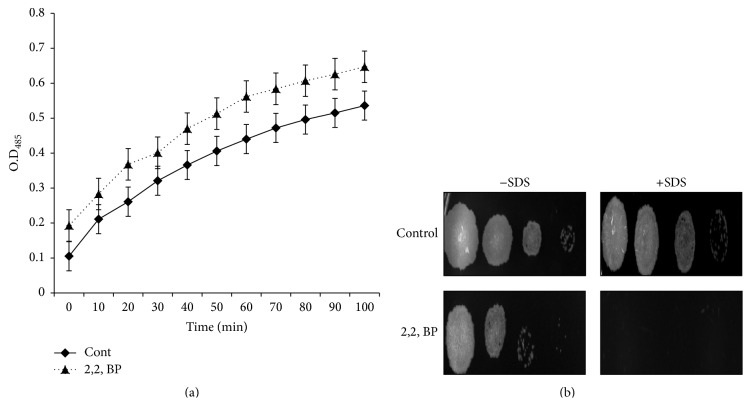
Effect of iron deprivation on cell membrane. (a) Nitrocefin membrane permeability assay for* M. smegmatis* cells grown in the absence (control) and the presence of 2,2, BP (35 *μ*g/mL). Means of O.D_485_ ± SD of three independent sets of experiments are depicted on *y*-axis with respect to time (minutes) on *x*-axis (*P* < 0.05). (b) Spot assay of* M. smegmatis* in the absence (control) and the presence of 2,2, BP (35 *μ*g/mL) and cell membrane perturbing agent (SDS) at 0.025%.

Cell membrane is one of the most significant barriers due to its complex lipid composition, hence a significant drug target of most of the commonly used anti-TB drugs [25]. We therefore explored the effect of iron deprivation on membrane, which in turn may affect the ability of the drug to permeate the cell membrane resensitizing the organism. For this, firstly, we perform the membrane permeability assay in response to iron deprivation by nitrocefin hydrolysis. Nitrocefin is a well-known chromogenic compound containing cephalosporin which is a class of *β*-lactam antibiotics. When this *β*-lactam ring of cephalosporin is hydrolyzed by *β*-lactamase enzyme, it turns from yellow to red color. When membrane becomes more permeable, nitrocefin is easily permitted to go inside the cell and get hydrolyzed which is measured as a change in absorbance at 486 nm. Thus, increased hydrolysis as depicted spectrophotometrically indicates enhanced membrane permeability. Interestingly, our data demonstrates (Figure 4(a)) that, in contrast to control cells, iron deprived cells showed more hydrolysis of nitrocefin significantly (*P* < 0.05). This implies that iron deprivation leads to enhanced membrane permeability which may be the causal reason for more drug intrusion inside the cell. This was further confirmed when the cells were spotted with the well-known membrane disrupting detergent SDS. Our results depict that, in the presence of SDS, iron deprived cells were hypersensitive in comparison to control cells (Figure 4(b)). Thus, we could establish that iron deprivation leads to perturbed membrane homeostasis and there could be a correlation between iron levels and lipid metabolism. This observation is also supported by the fact that there is an association of iron with GroEL1 protein which is required for fatty acid synthesis [26].

**Figure 5:**
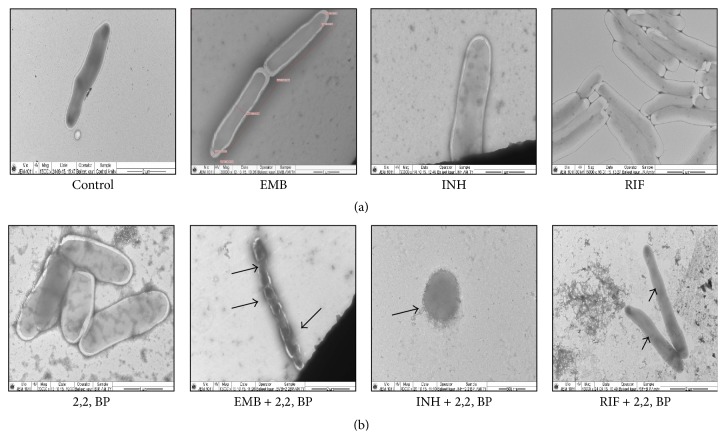
TEM images under iron deprivation. (a) TEM images for* M. smegmatis* cells grown in the absence (control) of 2,2, BP (35 *μ*g/mL) and drugs EMB, INH, and RIF with subinhibitory concentrations (62.5 ng/mL, 1 *μ*g/mL, and 62.5 ng/mL) alone showing smooth cell envelope. (b) TEM images for* M. smegmatis* cells grown in the presence of drugs (EMB, INH, and RIF) with subinhibitory concentrations (62.5 ng/mL, 1 *μ*g/mL, and 62.5 ng/mL) along with 2,2, BP (35 *μ*g/mL) showing tampered and elongated morphology.

That iron deprivation affects membrane integrity was further evident from the TEM images. To analyze any differences in the morphology or shape of mycobacterial cell envelope due to iron deprivation, TEM experiment was performed as described in Section 2. We observed that, in comparison to the cells without any drug (control), drugs (EMB, INH, and RIF) with subinhibitory concentrations (62.5 ng/mL, 1 *μ*g/mL, and 62.5 ng/mL), and iron deprivation (2,2, BP) alone which showed smooth cell envelope, iron deprived drugs treated cells showed tampered morphology as visualized by decreased cell wall thickness and distortion (Figure 5). Specifically, EMB and INH treated iron deprived cell structures were entirely distorted whereas in case of RIF cells structure showed filamentation, which is irregular or abnormal growth of bacteria in which they do not divide but continue to elongate [27]. These morphological changes are usually associated with exposure to antibiotics, nutrient depletion, and oxidative stress like ROS generation and DNA damage which could affect cell division or DNA replication process [28, 29].

### 3.5. Iron Deprivation Leads to Enhanced Passive Diffusion of Drug

**Figure 6:**
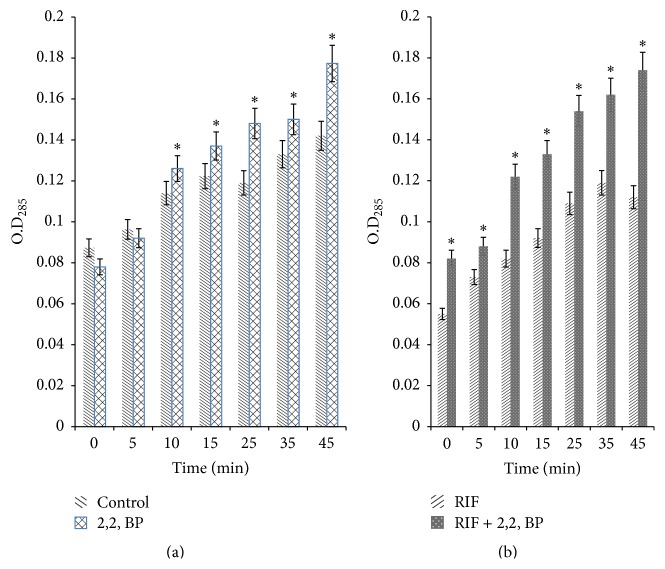
Passive diffusion in response to iron deprivation. (a) Passive diffusion displayed by extracellular concentrations of EtBr for* M. smegmatis* cells grown in the absence (control) and the presence of 2,2, BP (35 *μ*g/mL) as described in Section 2. Means of O.D_285_ ± SD of three independent sets of experiments are depicted on *y*-axis with respect to time (minutes) on *x*-axis (*∗* depicts significant difference with *P* < 0.05). (b) Passive diffusion displayed by extracellular concentrations of EtBr for* M. smegmatis* cells grown in RIF alone (control) and in the presence of 2,2, BP (35 *μ*g/mL) as described in Section 2. Means of O.D_285_ ± SD of three independent sets of experiments are depicted on *y*-axis with respect to time (minutes) on *x*-axis (*∗* depicts significant difference with *P* < 0.05).

Enhanced membrane permeability and disrupted morphology therefore prompted us to further explore the effect of iron deprivation on passive diffusion of drug across the cell membrane of* M. smegmatis*. This was achieved by estimating extracellular EtBr concentration in the absence and presence of iron deprived condition as described in Section 2. It is evident that (Figure 6(a)) after 45 min of incubation with 2,2, BP the supernatants showed increased extracellular EtBr concentration, implying enhanced (*P* value < 0.05) passive diffusion of the EtBr under iron deprivation. Next, we ascertain whether enhanced passive diffusion under iron deprivation also leads to enhanced diffusion of known anti-TB drug through membrane because of which now the same RIF can act more strongly as due to iron deprivation it can readily enter inside the cell. For this, the extracellular EtBr concentration was estimated for the cells treated with RIF in the absence and the presence of iron deprived condition. We observed that, in contrast to cells treated with RIF alone, iron deprived RIF treated cells showed increased extracellular EtBr concentration again implying enhanced passive diffusion (Figure 6(b)). These results reinforced the hypothesis that the effect of iron deprivation on* M. smegmatis* is linked with the perturbed cell membrane function.

### 3.6. Iron Is Indispensable to Sustain Genotoxic Stress

**Figure 7:**
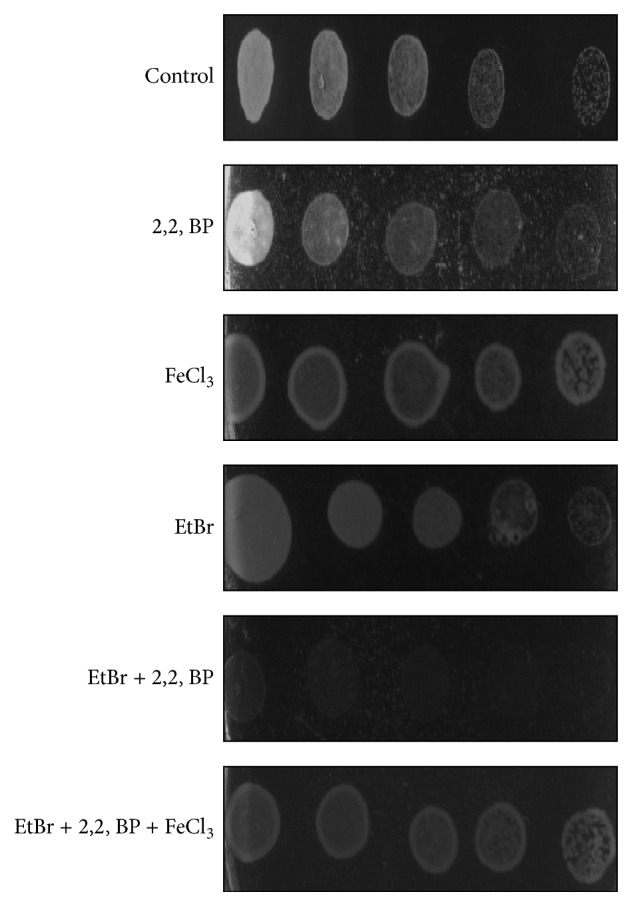
Indispensability of iron to sustain genotoxic stress. Spot assay of* M. smegmatis* in the absence (control) and the presence of 2,2, BP (35 *μ*g/mL) and DNA damaging agent (EtBr) at 13 *μ*g/mL.

*Mycobacteria* often reside inside the macrophages where they replicate and sustain the hostile environment. One of the immune responses, characterized by generation of NO (nitric oxide), and reactive element species (RNI and ROS) is mounted by the host to destroy the infectious agent. The RNI and ROS have high toxicity due to their ability to impose damages to biomolecules such as DNA, proteins, and lipids [30]. Mycobacterial genome contains high G + C content making its DNA highly susceptible to damage. In our study, we used EtBr, a well-known DNA damaging agent, at a concentration where there was no significant growth defect confirming the presence of functional DNA repair machinery. Interestingly, we observed that iron deprived cells were hypersensitive to EtBr as compared to control cells suggesting that iron deprivation leads to abrogate DNA repair machinery (Figure 7). Furthermore, to confirm the indispensability of iron to cope genotoxicity, we supplement the media with iron FeCl_3_ and observed that the sensitivity of EtBr could be rescued (Figure 7). This confirms that the presence of iron is crucial for survival against genotoxic stress and that iron deprivation is hindering the DNA repair mechanism; however, the precise mechanism still needs to be validated. Our results also corroborate well with the morphological changes we observed under iron deprivation which is also known to be associated with DNA damage affecting cell division or DNA replication process (see above).

### 3.7. Drug Susceptibility Remains Unaltered in Alkaline pH

**Figure 8:**
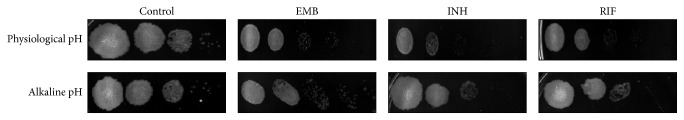
Drug susceptibility assay under alkaline pH. Spot assay of* M. smegmatis* in the absence and the presence of drugs EMB, INH, and RIF (62.5 ng/mL, 1 *μ*g/mL, and 62.5 ng/mL) at physiological and alkaline pH 10.

As a known matter of fact alkaline pH mimics iron deficiency. This is particularly evident from the fact that at alkaline pH most of the available iron is present in insoluble ferric form thus representing iron deprived condition [4]. Thus, enhanced drug susceptibilities of known anti-TB drugs observed under iron deprivation in the present study necessitated testing of similar drug susceptibilities in alkaline condition which is present in human at several niches. To test this, we performed similar spot assays under alkaline pH in presence of all the above tested anti-TB drugs (EMB 62.5 ng/mL, INH 1 *μ*g/mL, and RIF 62.5 ng/mL). Interestingly, our results as depicted by spot assays do not show any difference between the cells grown at either physiological or alkaline pH (Figure 8). This suggests that iron and pH regulatory circuits are not governed by common regulators in mycobacteria.

## 4. Conclusion

Taken together, our results demonstrate that iron deprivation of* M. smegmatis* cells affects cellular membrane integrity, which in turn presumably allows faster entry of drugs leading to enhanced drug sensitivity of the cells. The possibility of coregulation of MDR, lipid biosynthesis, and iron acquisition genes through common regulators may also exist, as has already been observed in several instances, but needs further validation. In conclusion, changes in the drug susceptibility of mycobacteria due to iron represent a well-regulated new mechanism that merits a closer look.
